# Current Trends and Research Challenges Regarding “Preparation for Oxidative Stress”

**DOI:** 10.3389/fphys.2017.00702

**Published:** 2017-09-25

**Authors:** Daniel C. Moreira, Marcus F. Oliveira, Lara Liz-Guimarães, Nilda Diniz-Rojas, Élida G. Campos, Marcelo Hermes-Lima

**Affiliations:** ^1^Departamento de Biologia Celular, Universidade de Brasília Brasilia, Brazil; ^2^Área de Morfologia, Faculdade de Medicina, Universidade de Brasília Brasilia, Brazil; ^3^Instituto de Bioquímica Médica Leopoldo de Meis, Universidade Federal do Rio de Janeiro Rio de Janeiro, Brazil; ^4^Departamento de Genética e Morfologia, Universidade de Brasília Brasilia, Brazil

**Keywords:** antioxidant, biochemical adaptation, estivation, hypoxia, oxidative stress, reactive oxygen species

## Abstract

Survival under stress, such as exposure to hypoxia, anoxia, freezing, dehydration, air exposure of water breathing organisms, and estivation, is commonly associated to enhanced endogenous antioxidants, a phenomenon coined “preparation for oxidative stress” (POS). The regulation of free radical metabolism seems to be crucial under these selective pressures, since this response is widespread among animals. A hypothesis of how POS works at the molecular level was recently proposed and relies on two main processes: increased reactive species production under hypoxia, and activation of redox-sensitive transcription factors and signaling pathways, increasing the expression of antioxidants. The present paper brings together the current knowledge on POS and considers its future directions. Data indicate the presence of POS in 83 animal species (71.6% among investigated species), distributed in eight animal phyla. Three main research challenges on POS are presented: (i) to identify the molecular mechanism(s) that mediate/induce POS, (ii) to identify the evolutionary origins of POS in animals, and (iii) to determine the presence of POS in natural environments. We firstly discuss the need of evidence for increased RS production in hypoxic conditions that underlie the POS response. Secondly, we discuss the phylogenetic origins of POS back 700 million years, by identifying POS-positive responses in cnidarians. Finally, we present the first reports of the POS adaptation strategy in the wild. The investigation of these research trends and challenges may prove useful to understand the evolution of animal redox adaptations and how they adapt to increasing stressful environments on Earth.

## Introduction

Animals are naturally submitted to environmental stresses that act as selective pressures leading to fixation of many behavioral, physiological, and biochemical adaptations. One situation that many animals endure is the periodic reduction in oxygen availability, which can last from hours to days/weeks. This occurs in animals during several natural events, including exposure to hypoxic/anoxic environments, exposure to freezing, or severe dehydration (which resemble ischemia), air exposure of water breathing organisms and estivation. These conditions are termed as “low oxygen stress” (Hermes-Lima et al., 2015).

Several biochemical adaptations, including metabolic depression, use of anaerobic pathways, epigenetic modifications, and changes in redox metabolism are conserved among many animal species that tolerate low oxygen stress (Staples and Buck, 2009; Storey and Storey, 2012; Biggar and Storey, 2015; Storey, 2015). In the last 25 years, researchers have been studying the role of redox metabolism in the survival machinery of animals under low oxygen stress and estivation. It was observed that many animal species from eight phyla (including vertebrates and invertebrates) upregulate endogenous antioxidant levels during low oxygen stress (Moreira et al., 2016). Phenotypically, studies from many laboratories have shown increases in catalase, superoxide dismutases, and glutathione peroxidases activities, and also in the levels of reduced glutathione (GSH), under stress conditions. The biological phenomenon of antioxidant upregulation in response to low oxygen availability is referred to as “preparation for oxidative stress” (POS; Hermes-Lima et al., 1998, 2001; Hermes-Lima and Zenteno-Savín, 2002).

The first observation of such phenomenon was an 183% increase in catalase activity in muscle samples from garter snakes (*Thamnophis sirtalis*) upon 5 h freezing exposure (Hermes-Lima and Storey, 1993). Further investigations showed that many other animal species, from cnidarians to vertebrates, enhance their endogenous antioxidants when exposed to different low oxygen stress conditions (Welker et al., 2013; Moreira et al., 2016). The reports of animals expanded from just six species in the 1990's (Storey, 1996; Hermes-Lima et al., 1998) to 69 species exhibiting POS as a general response to low oxygen stresses (Moreira et al., 2016). The present article updates the list to 83 animal species (see section The Prevalence of POS in 8 Animal Phyla—A Brief Update). The widespread distribution of POS in the animal kingdom indicates that the regulation of free radical metabolism is crucial under such selective pressures posed by abiotic stresses.

In the present article, we discuss the current trends and research challenges on redox adaptation in animals exposed to low oxygen stress. We report the state of the art as well as the key research challenges regarding the POS phenomena.

### Reviewing the biochemical model for POS

At the time POS was coined, in 1990's, the term “preparation” referred to the build-up of antioxidant defenses in advance to the upcoming “oxidative stress” that would occur only when O_2_ availability is resumed. Thus, animals would prepare themselves, in the absence of an apparent oxidative stimulus, avoiding the reoxygenation stress. For a long time, the identity of the molecular signal to trigger the boost in antioxidant defenses was unknown and speculated to be a “non-radical” signal (Hermes-Lima and Storey, 1993). This was mainly due to the premise that reactive (oxygen/nitrogen) species (RS) production would necessarily decrease under hypoxia. Because the only known condition to activate endogenous antioxidants was oxidative stress (and increased RS formation), the POS phenomenon remained without a biochemical mechanism of activation.

This changed in the comparative biology field around 2005–2007, when a growing number of studies considered that RS formation could actually increase during low oxygen availability (Bickler and Buck, 2007; Lushchak and Bagnyukova, 2007). Despite of the evidence (e.g., Chandel et al., 1998; Guzy et al., 2005), researchers remained resistant to accept the idea of elevated RS production during hypoxia (Clanton, 2005). Currently, it is well-known that oxygen deprivation itself, and not only the reoxygenation event, poses an oxidative insult to cells (Waypa et al., 2016). Based on data from comparative biology, as well as from medical science, a biochemical model involving redox signaling was recently proposed to explain the boost in antioxidant defenses during low oxygen stress in hypoxia-tolerant species (Hermes-Lima et al., 2015). Regardless of the current understanding that hypoxia may also be a condition of oxidative stress, besides reoxygenation, the term “preparation” is still used because it became widely used in the literature, especially in hypoxia tolerance research.

**Figure 1:**
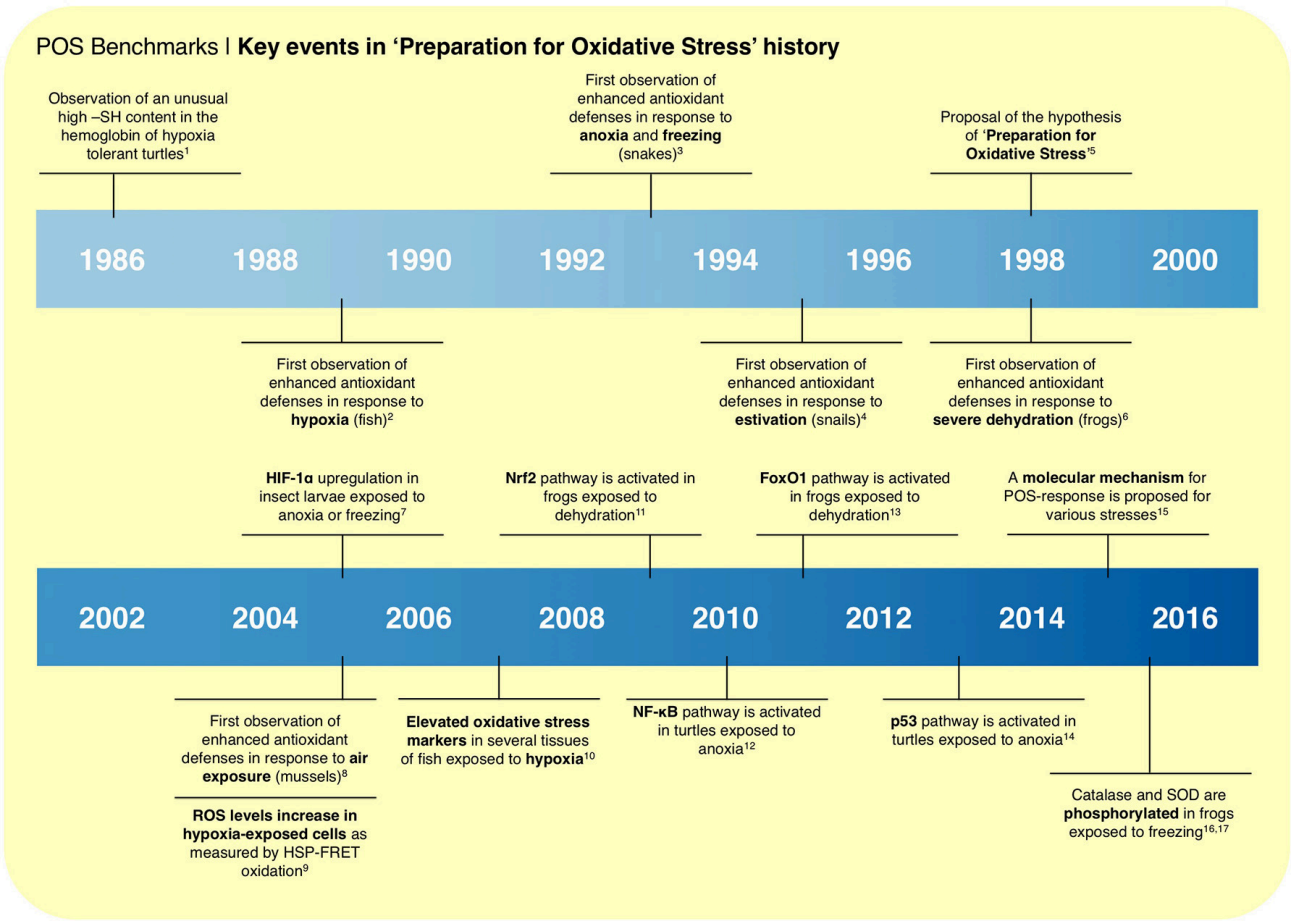
Milestones in “preparation for oxidative stress” research, from the first observation that hypoxia-tolerant turtles have unusual hemoglobin with high thiol content in 1986; through the many observations of increased endogenous antioxidants levels in animals exposed to hypoxia, anoxia, freezing, estivation, severe dehydration, and air exposure. Key events include the observations of redox-sensitive transcription factors activation in animals exposed to low oxygen stresses and the post-translational control antioxidant enzymes by phosphorylation. “Preparation for oxidative stress” was coined in 1998. In 2015, a biochemical model was proposed to explain the widespread observation of enhanced antioxidant defenses in animals exposed to low oxygen stresses and estivation. Superscript letters refer to: ^1^(Reischl, 1986); ^2^(Radi et al., 1988); ^3^(Hermes-Lima and Storey, 1993); ^4^(Hermes-Lima and Storey, 1995); ^5^(Hermes-Lima et al., 1998); ^6^(Hermes-Lima and Storey, 1998); ^7^(Morin et al., 2005); ^8^(Almeida et al., 2005); ^9^(Guzy et al., 2005); ^10^(Lushchak and Bagnyukova, 2007); ^11^(Malik and Storey, 2009); ^12^(Krivoruchko and Storey, 2010); ^13^(Malik and Storey, 2011); ^14^(Zhang et al., 2013); ^15^(Hermes-Lima et al., 2015); ^16^(Dawson et al., 2015); ^17^(Dawson and Storey, 2016).

**Figure 2:**
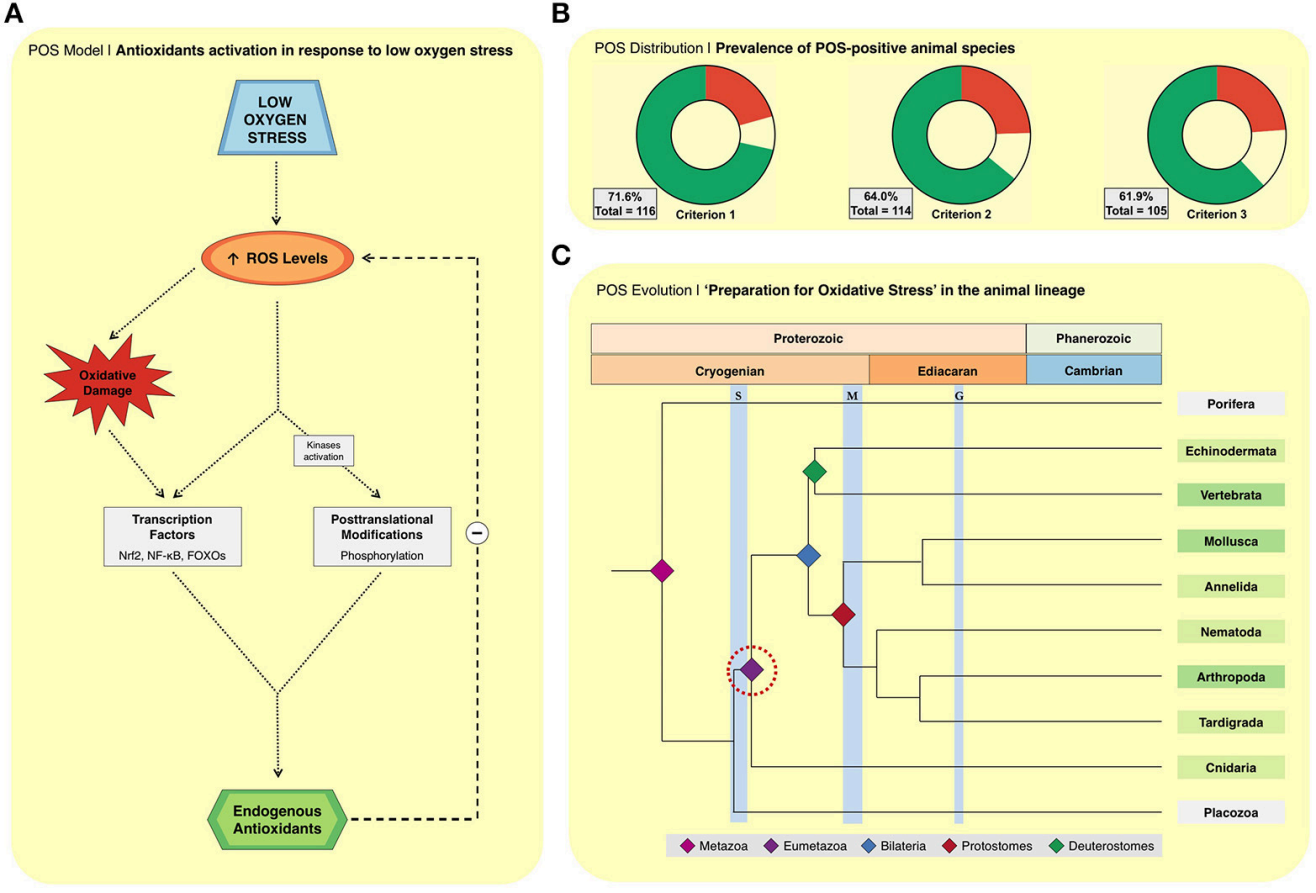
**(A)** The biochemical model of how antioxidant defenses are enhanced in response to low oxygen stress or estivation (i.e., “preparation for oxidative stress;” Hermes-Lima et al., 2015). This model assumes that: (i) a temporary increase in reactive oxygen species (ROS) steady-state levels occurs during exposure to low oxygen stress (air exposure, anoxia, freezing, hypoxia, and dehydration) or early estivation; (ii) excessive ROS lead to redox imbalance; (iii) sustained redox imbalance results in physiological oxidative damage, activation of redox-sensitive transcription factors (FoxOs, NF-κB, and Nrf2), and activation of protein kinases, all leading to upregulation of the endogenous antioxidant apparatus. **(B)** Prevalence of animal species classified by criterion 1, 2, and 3 as described by Moreira et al. (2016) as positive (green), neutral (yellow) and negative (red) for low oxygen stresses and estivation combined (all species). Herein we added new studies and species not reported in the previous publication (Moreira et al., 2016). The 14 species added in this study are *Astronotus ocellatus* (Marcon, 1996); *Bunodosoma cangicum* (Abujamara et al., 2014); *Catla catla* (Singh et al., 2016); *Colossoma macropomum* (Marcon, 1996); *Crepipatella dilatata* (Cubillos et al., 2016); *Danio rerio* (Feng et al., 2016); *Larimichthys crocea* (Wang et al., 2017); *Neohelice granulata* ((Geihs et al, 2016); *Pandalus borealis* (Dupont-Prinet et al., 2013; Pillet et al., 2016); *Pelteobagrus fulvidraco* (Yang et al., 2014); *Pelteobagrus vachelli* (Zhang et al., 2016); *Plectus murrayi* (Adhikari et al., 2009); *Reinhardtius hippoglossoides* (Pillet et al., 2016); *Scapharca inaequivalvis* (Foschi et al., 2012). **(C)** Illustrative topological drawing of metazoan radiation for 10 animal lineages (Erwin et al., 2011; Erwin, 2015). Phyla that comprise POS-positive species are shown in green. Those with more studied species are in darker green (Arthropoda, Mollusca, and Chordata). Groups with fewer studied species are shown in lighter green. Phyla without evidence for POS are shown in gray. The red circle indicates the most ancient evidence of POS, considering it as a monophyletic characteristic.

Several events mark the transitions from the early observations of enhanced endogenous antioxidants (Figure 1), to the current molecular model in the comparative biology field (Figure 2A). This model is the result of the compilation of different observations from the literature: (i) RS formation may increase in biological systems exposed to oxygen deprivation; (ii) several hypoxia-tolerant animals show signs of oxidative stress during hypoxia; (iii) the expression of antioxidant defenses is controlled by redox-sensitive transcription factors, such as Nrf2, NF-κB, and FOXOs; and (iv) the observations that these same transcription factors are activated in hypoxia-tolerant animals exposed to low oxygen stress (Hermes-Lima et al., 2015). In this context, RS (and oxidative damage by-products) would act as signaling molecules that activate redox-sensitive transcriptional factors, and then antioxidant enzymes (Welker et al., 2013; Hermes-Lima et al., 2015; Figure 2A). Such transcription factors are well-known to respond to oxidative challenges in many biological systems (Sena and Chandel, 2012; Scotcher et al., 2013; Espinosa-Diez et al., 2015; Klotz et al., 2015). Another consequence of oxidative challenges is the activation of protein kinases that targets catalase, GPX and SOD (Cao et al., 2003a,b) and decreased phosphatase activity (Machado et al., 2017). Recently, it was shown that phosphorylation activates MnSOD (Dawson et al., 2015) and catalase (Dawson and Storey, 2016) in wood frogs (*Rana sylvatica*) exposed to freezing. Thus, the same cellular pathways involved in the oxidative stress response in conventional models (e.g., mice and mammalian cells) would operate in animals that are naturally tolerant to severe O_2_ deprivation. The redox imbalance during hypoxia would act as a hormetic signal, boosting antioxidant defenses, which would mitigate the expected RS burst during reoxygenation. Such effect resembles that of mammalian cardiac pre-conditioning (Zhou et al., 1996) or mild oxidative challenges in working muscle, in which exposure to mild oxidative stress improves cellular function and preservation mechanisms (Nikolaidis et al., 2012).

## First challenge—are RS levels increased in hypoxia and the triggers for POS?

Increased RS production under hypoxia has been measured in tissues and cells from hypoxia sensitive model organisms (e.g., probes or oxidation products; reviewed in Hermes-Lima et al. (2015) and Waypa et al. (2016). For instance, RS production in response to acute hypoxia increases in endothelial cells (Hernansanz-Agustín et al., 2014). Moreover, the kinetics of such RS overproduction is in the scale of minutes and depends on the degree of hypoxia (Hernansanz-Agustín et al., 2014). When the experiment was performed using endothelial cells without a functional mitochondria there was no rise in RS formation, indicating that such burst in RS emanates from mitochondria (Hernansanz-Agustín et al., 2014). This kind of evidence, however, is lacking for hypoxia tolerant animals that exhibit the POS-response (i.e., antioxidant upregulation) to low oxygen stress. Thus, the first scientific challenge in POS research is the verification of the hypothesis that mitochondrial RS formation increases in animal species that enhance endogenous antioxidants during hypoxic challenges.

While mitochondrial superoxide radical (${\text{O}}_{2}^{•-}$), and H_2_O_2_, are candidate initiators in our model (Hermes-Lima et al., 2015), other reactive species, as well as other cellular sources of RS, may also play a role. Nitric oxide, for example, may reversibly inhibit components of the mitochondrial respiratory chain and alter the rate of RS formation (Cleeter et al., 1994). Also, mitochondrial RS production is highly regulated by different factors, including the electron flux, the magnitude of the protonmotive force, the oxygen and substrate availability, and the NADH/NAD^+^ ratio in the matrix (Boveris and Chance, 1973; Korshunov et al., 1997; Nishikawa et al., 2000; Miwa et al., 2003; Yu et al., 2006; Aon et al., 2010; Ronchi et al., 2013).

In contrast to the numerous reports of enhanced endogenous antioxidants in response to low oxygen stress, there is no direct evidence that the proposed increase in mitochondrial RS formation actually occurs in species that present POS as a response. However, scattered evidence indicates the occurrence of redox imbalance and oxidative stress during hypoxia, as several oxidative stress markers are elevated during low oxygen stress (Hermes-Lima et al., 2015). To our knowledge, there are three studies that quantified RS production in oxygen deprived non-conventional models. These studies, however, do not indicate any increase in RS production under hypoxia exposure for those specific time-points. In one study, cerebral RS formation, determined as salicylate hydroxylation in cerebrospinal fluid, presented a trend for decrease in the first hour and significantly decreased to nearly zero within 4 h of anoxia exposure in *Trachemys scripta* turtles (Milton et al., 2007). In another study, RS formation was evaluated in the marine platyhelminth *Macrostomum lignano* exposed to near-anoxia for 1.5 h. Superoxide levels, determined as DHE staining, were unaffected by anoxia exposure (Rivera-Ingraham et al., 2013a). However, oxidant generation (assessed by C-H_2_DFFDA staining) in anoxia-exposed worms decreased by 78% when compared to normoxic animals (Rivera-Ingraham et al., 2013a). The same probes (DHE and C-H_2_DFFDA) were used to measure RS levels in the gills of blue mussels under near-anoxia for 48 and 72 h. The results from both probes indicated a reduction in RS formation (Rivera-Ingraham et al., 2013b). Together, these studies indicate either a reduction or maintenance of oxidant levels *in vivo* in anoxia/hypoxia challenged animals. Thus, the proposed increase in RS generation was not observed in POS-responsive species yet.

## Second challenge—occurrence and evolution of POS in the animal kingdom

Recently, we mapped the prevalence of POS in various animal species that had their antioxidant response assessed under low oxygen stress or estivation (Moreira et al., 2016). To do so, we developed three criteria to classify species as positive, neutral or negative. First criterion: species are classified as positive if there is at least one upregulation event of antioxidant defense, regardless of other alterations. Second criterion: species are classified as positive if there is at least one upregulation above a 50% threshold; or as negative if there is no upregulation event, but downregulation events above a 25% threshold. Third criterion: species are classified as positive only if there are more events of upregulation (above 50%) in comparison to downregulation (above 25%) within a tissue, if the number of up and downregulation matches, the species is classified as neutral. Applying the first criterion, in a list of 102 species from eight animal phyla, there were observations of increased levels of antioxidant enzymes in 68% of these species (69 out of 102) under low oxygen stress or estivation (Moreira et al., 2016). This first glance at the responses to low oxygen stress and estivation of a great number of animal species—vertebrates and invertebrates—evidenced the recurrent nature of POS phenomena. Such high prevalence of animals that spend resources on endogenous antioxidants in such energy-limited situations indicates a key adaptive role of POS. Below; we update this list to 83 species that fit the POS criteria.

### The prevalence of POS in 8 animal Phyla—A brief update

Because new studies have been published and a few other works were missed, the percentages of POS-positive species changed in comparison to our last analysis (Moreira et al., 2016). The current list presents 116 animal species, 83 classified as POS-positive in criteria 1 (72%; Figure 2B). We are, however, more focused on those species considered for the most restrictive criteria 3 (*n* = 105, 62% POS-positive). The prevalence of POS-positive species tended to increase in comparison to the previous values (Moreira et al., 2016). For instance, all studied species under estivation or severe dehydration are classified as POS-positive according to criterion 3. In the case of freezing, anoxia, and air exposure, the prevalence of POS-positive species is 83, 67, and 60%, respectively. The prevalence of POS-positive species is lowest in animals exposed to hypoxia (47%), which is the stress with most species studied (*n* = 70) and also with highly variable protocols. Thus, the prevalence of POS-positive species varies depending on the stress to which animals are exposed.

From a phylogenetic point of view, it is noteworthy that from the 65 animal species currently classified as POS-positive (in criterion 3), 57 of them were from only three phyla: Mollusca, Arthropoda, and Chordata. Mollusks had 25 species analyzed and 14 of them (56%) are considered POS-positive. POS-positive arthropods were 58%. In the case of Chordata, 64% of the analyzed species are POS-positive. Moreover, the presence of the POS mechanism in cnidarians is of special relevance in terms of evolution because the phylogenetic line of these animals started to evolve hundreds of millions of years ago (Erwin, 2015). Noteworthy, the expression of several key components of the hypoxia tolerance machinery and POS-response are present in cnidarians. Indeed, HIF-1 and other redox-sensitive transcription factors crucial for the POS-response—FoxO, Nrf2, and NF-κB—have been found in several cnidarians (Sullivan et al., 2007; Meyer et al., 2009; Baumgarten et al., 2015; Malafoglia et al., 2016). Since most molecular mediators for POS seems to be working in cnidarians, it is possible that this process is controlled in hypoxia-tolerant species of this Phylum by a mechanism very similar to that we proposed to major bilaterian groups.

### Animal evolution and POS

To place the origin of the “POS phenotype” in animal evolution is the second challenge in POS research. The POS-response was observed in cnidarians, which is an ancient animal group. Teixeira et al. (2013) reported the activation of antioxidant defenses in the octocoral *Veretillum cynomorium* as a consequence of aerial exposure. In another study, Abujamara et al. (2014) reported a significant increase in SOD activity upon exposure of anemones *Bunodosoma cangicum* to hypoxia. Since POS is present in many bilaterian groups and in cnidarians, it is reasonable that it was already present in the last common ancestor of both groups. It is estimated that the divergence of Cnidaria and Bilateria happened about 700 million years ago (Ma), before the Marinoan glaciation (Figure 2C; Erwin et al., 2011; Shu et al., 2014; Erwin, 2015). Moreover, even though there are unequivocal fossil records of cnidarians dated from the Fortunian epoch of the Cambrian, there is much uncertainty whether other fossils from the late Ediacaran could be identified as cnidarian (Shu et al., 2014). In any case, many more studies are needed on low oxygen stress and POS-response in cnidarians since there is only two POS-positive species in this Phylum. It would also be valuable to assess alterations of the redox metabolism in hypoxia-tolerant species from basal phyla, such as Porifera.

## Third challenge—assessment of POS in the wilderness

The third scientific challenge for POS research is to verify whether the POS-phenotype is present in animals in their natural environment in contrast to animals studied in the laboratory. Most published studies were conducted in the laboratory under artificially controlled conditions, being in some cases not ecologically relevant. Our preliminary results show that when the frogs *Pleurodema diplolistris* and *Proceratophrys cristiceps* naturally estivate under dry riverbeds of a semi-arid region in Northeast Brazil there is a 50–75% increase in muscular catalase activity—in comparison with active animals in the same site, but in rainy season (Moreira et al., 2017). In another example of POS under natural settings, mussels *Brachidontes solisianus* increased their whole-body GSH levels in response to air exposure for 4 h compared to animals underwater in Southern Brazil (Sabino et al., 2015). These examples indicate that POS may occur in the wild. However, studies with other animal species are needed to show more evidence that the POS-phenotype is not restricted to laboratory conditions, and that it indeed happens in nature.

## Final statements

As stated in On the Origin of Species: “*Climate plays an important part in determining the average numbers of a species, and periodical seasons of extreme cold or drought, I believe to be the most effective of all checks*” (Darwin, 1859). That is, the environment and its changing conditions have a critical role in shaping life forms, acting as major selective pressures. As discussed herein, the regulation of redox metabolism seems to be crucial for adaptation. Preparation for oxidative stress is a phenomenon that happens in 62–72% of animal species tolerant to low oxygen stress or estivation that were analyzed for the antioxidant regulation. This prevalence is also dependent on the animal taxon and the kind of stress-response. Future studies will likely change the prevalence of POS in the animal kingdom—so far, 116 species were analyzed.

It has been a long way from the initial proposals of this redox adaptation strategy (Reischl, 1986; Hermes-Lima et al., 1998) to the current molecular model (Welker et al., 2013; Hermes-Lima et al., 2015). The current investigative path is, first, to identify the molecular trigger of POS, possibly increased superoxide/H_2_O_2_ formation. The redox imbalance caused by such increase during low oxygen stress may activate a protective response against a forthcoming challenge in reoxygenation—fitting into the hormesis concept (Costantini, 2014). Second, it is crucial to place POS in the history of life. We suggest that POS could have been present at least ~700 Ma, in the last common ancestor of cnidarians and bilaterians (Figure 2C), a period where oxygen was 1–3% of the present levels (Erwin, 2015). Finally, it is a priority to determine whether POS happens in the wild. Preliminary determinations in estivating frogs and air-exposed mussels suggest the answer is “yes,” however, more species must be studied.

There are other issues related to POS that deserve investigation. For example, the occurrence of hypoxia in freshwater and coastal environments is an expanding threat to aquatic life (Vaquer-Sunyer and Duarte, 2008). Deeper knowledge about how POS works and evolved may prove useful to understand how ecosystems will respond to this growing global problem. Would it induce selective pressure favoring POS-positive organisms? This represents a look onto how POS would affect animals in the future.

## Author contributions

DM, MO, ÉC, and MH contributed to the eco-physiological and molecular concepts of the work. ND, LL, and MH contributed with the evolutionary ideas. All authors reviewed the bibliography and discussed the overall data/concepts. DM and MH drafted the manuscript, which was reviewed and approved by all authors.

### Conflict of interest statement

The authors declare that the research was conducted in the absence of any commercial or financial relationships that could be construed as a potential conflict of interest.
