# Corrigendum: Current Trends and Research Challenges Regarding “Preparation for Oxidative Stress”

**DOI:** 10.3389/fphys.2018.00950

**Published:** 2018-07-13

**Authors:** Daniel C. Moreira, Marcus F. Oliveira, Lara Liz-Guimarães, Nilda Diniz-Rojas, Élida G. Campos, Marcelo Hermes-Lima

**Affiliations:** ^1^Departamento de Biologia Celular, Universidade de Brasília, Brasilia, Brazil; ^2^Área de Morfologia, Faculdade de Medicina, Universidade de Brasília, Brasilia, Brazil; ^3^Instituto de Bioquímica Médica Leopoldo de Meis, Universidade Federal do Rio de Janeiro, Rio de Janeiro, Brazil; ^4^Departamento de Genética e Morfologia, Universidade de Brasília, Brasilia, Brazil

**Keywords:** antioxidant, biochemical adaptation, estivation, hypoxia, oxidative stress, reactive oxygen species

In the original article, there was an error. In the Introduction text, the word “oxygen” was used instead of “oxidative.” The correct term is “preparation for oxidative stress.”

A correction has been made to the Introduction, second paragraph:

Several biochemical adaptations, including metabolic depression, use of anaerobic pathways, epigenetic modifications, and changes in redox metabolism are conserved among many animal species that tolerate low oxygen stress (Staples and Buck, 2009; Storey and Storey, 2012; Biggar and Storey, 2015; Storey, 2015). In the last 25 years, researchers have been studying the role of redox metabolism in the survival machinery of animals under low oxygen stress and estivation. It was observed that many animal species from eight phyla (including vertebrates and invertebrates) upregulate endogenous antioxidant levels during low oxygen stress (Moreira et al., 2016). Phenotypically, studies from many laboratories have shown increases in catalase, superoxide dismutases, and glutathione peroxidases activities, and also in the levels of reduced glutathione (GSH), under stress conditions. The biological phenomenon of antioxidant upregulation in response to low oxygen availability is referred to as “preparation for oxidative stress” (POS; Hermes-Lima et al., 1998, 2001; Hermes-Lima and Zenteno-Savín, 2002).

The authors apologize for this error and state that this does not change the scientific conclusions of the article in any way.

The original article has been updated.

## Conflict of interest statement

The authors declare that the research was conducted in the absence of any commercial or financial relationships that could be construed as a potential conflict of interest.
